# Improvement of the therapeutic capacity of insulin-producing cells trans-differentiated from human liver cells using engineered cell sheet

**DOI:** 10.1186/s13287-020-02080-0

**Published:** 2021-01-06

**Authors:** Yu Na Lee, Hye-Jin Yi, Eun Hye Seo, Jooyun Oh, Song Lee, Sarah Ferber, Teruo Okano, In Kyong Shim, Song Cheol Kim

**Affiliations:** 1 Asan Institute for Life Sciences, Asan Medical Center, University of Ulsan College of Medicine, Seoul, Republic of Korea; 2 Sheba Regenerative Medicine, Stem Cells and Tissue Engineering Center, Sheba Medical Center, Tel-Hashomer, Israel; 3 Institute of Advanced Biomedical Engineering and Science, Tokyo Women’s Medical University, Tokyo, Japan; 4 Cell Sheet Tissue Engineering Center, Department of Pharmaceutics and Pharmaceutical Chemistry, University of Utah, Salt Lake City, USA; 5 Department of Surgery, Asan Medical Center, University of Ulsan College of Medicine, Seoul, Republic of Korea

**Keywords:** Liver cell, Insulin-producing cell, Diabetics, Cell sheet, Transplantation

## Abstract

**Background:**

Although pancreatic islet transplantation therapy is ideal for diabetes patients, several hurdles have prevented it from becoming a standard treatment, including donor shortage and low engraftment efficacy. In this study, we prepared insulin-producing cells trans-differentiated from adult human liver cells as a new islet source. Also, cell sheet formation could improve differentiation efficiency and graft survival.

**Methods:**

Liver cells were expanded in vitro and trans-differentiated to IPCs using adenovirus vectors carrying human genes for *PDX1*, *NEUROD1*, and *MAFA*. IPCs were seeded on temperature-responsive culture dishes to form cell sheets. Differentiation efficiency was confirmed by ß cell-specific gene expression, insulin production, and immunohistochemistry. IPC suspension was injected by portal vein (PV), and IPC sheet was transplanted on the liver surface of the diabetic nude mouse. The therapeutic effect of IPC sheet was evaluated by comparing blood glucose control, weight gain, histological evaluation, and hepatotoxicity with IPC injection group. Also, cell biodistribution was assessed by in vivo/ex vivo fluorescence image tagging.

**Results:**

Insulin gene expression and protein production were significantly increased on IPC sheets compared with those in IPCs cultured on conventional culture dishes. Transplanted IPC sheets displayed significantly higher engraftment efficiency and fewer transplanted cells in other organs than injected IPCs, and also lower liver toxicity, improved blood glucose levels, and weight gain. Immunohistochemical analyses of liver tissue revealed positive staining for PDX1 and insulin at 1, 2, and 4 weeks after IPC transplantation.

**Conclusions:**

In conclusion, cell sheet formation enhanced the differentiation function and maturation of IPCs in vitro. Additionally, parameters for clinical application such as distribution, therapeutic efficacy, and toxicity were favorable. The cell sheet technique may be used with IPCs derived from various cell sources in clinical applications.

## Background

Diabetes causes multiple systemic complications such as blindness, kidney failure, heart attacks, stroke, and diabetic neuropathy, which reduce life expectancy and impose enormous health costs worldwide [1]. Insulin therapy is the most effective method for reducing hyperglycemia. However, general insulin therapy cannot prevent severe hypoglycemia and long-term complications [2–4]. Strict glycemic control has been emphasized in the management of diabetes [5, 6]. Currently, pancreas and islet-cell transplantations are the only treatment modalities to prevent late complications of insulin-dependent diabetes. Islet transplantation is comparatively simple and non-invasive, in contrast to pancreas transplantation. Shapiro et al. examined seven patients successfully treated for insulin dependence for 1 year after islet implantation. Over the long follow-up, transplanted islets showed progressive loss of function, and many patients required insulin therapy [7]. Many limitations must be overcome for successful islet transplantation. Developing autologous insulin-producing cells (IPCs) from patients is an ideal solution to overcome the current islet transplantation limitations, including the shortage of donors and immune reactions of the allograft [8].

Use of liver cells from the liver has attracted considerable attention. These cells are considered a good source for trans-differentiation into pancreatic cells because they share a common developmental origin with the pancreas and are free from the safety risks associated with stem cells [9, 10]. Recent studies demonstrated that adult cells or stem cells can be induced to the pancreatic lineage by overexpressing pancreatic and duodenal homeobox 1 (PDX1), which is crucial for pancreatic organogenesis and beta cell function [11, 12]. Additionally, various transcription factors play important roles in the differentiation and maturation of IPCs, including neurogenin-3 (NGN3), neuronal differentiation 1 (NEUROD1), and MAFA [13]. Interestingly, Berneman-Zeitouni et al. showed that the temporal and hierarchical combination of three pancreatic transcription factors, PDX1, paired box 4 (PAX4), and v-maf musculoaponeurotic fibrosarcoma oncogene family, protein A (MAFA), enhanced mature β cell-like characteristics [14]. The development of a variety of factors has resulted in improvements in differentiation capability that includes insulin gene expression and in vivo insulin secretion in vitro.

Cell sheets are harvested from temperature-responsive culture dishes by inducing temperature change, which enables them to maintain cell-cell connections and adhesive proteins on their basal side. This facilitates attachment to target sites in other tissues. Cell sheets have the potential to deliver a large number of cells to the desired organ without the loss of transplanted cells [15]. Clinically, pancreatic islet transplantation is conducted via the intraportal route [16]. When islets are administered directly through the portal vein, many cells engraft in the liver through smaller venules, enabling the liver to supply blood with near-physiological insulin delivery [17]. However, concerns with intraportal islet infusion include procedure-related complications, bleeding, hepatic hypertension, and thrombosis [16]. Particularly, the cell transplantation efficiency is quite low and the instant blood-mediated inflammatory reaction causes rapid islet loss [18, 19].

The purpose of the present study was to investigate the effect of IPC sheets trans-differentiated from human liver cells in enhancing the differentiation efficiency and graft survival of IPCs. We compared pancreatic lineage-related gene expression and insulin secretion on IPC sheets to those in IPCs cultured on conventional culture dishes. To assess the therapeutic efficacy, biodistribution, and safety of IPC sheets, we compared two different techniques for transplantation into the liver: a single injection of IPCs through the portal vein and transplantation of the IPC sheet onto the liver surface.

## Methods

### Isolation of human liver cells

Adult liver cells were isolated and cultured as previously described [11, 14]. This study was carried out according to the guidelines and with the approval of the Institutional Review Board of Asan Medical Center (IRB number: 2014-1182 Seoul, Republic of Korea). Liver tissue was obtained from donors undergoing abdominal surgery; informed consent was obtained from the patients for this procedure. In the case of non-diabetic patients and patients with type 2 diabetes, liver tissues were obtained with consent from patients who underwent pancreatic surgery due to benign pancreatic disease. In the case of patients with type 1 diabetes, liver tissue was obtained with consent from patients who underwent abdominal surgery for pancreatic transplantation. The selection criteria for liver tissue biopsy participants included patients undergoing pancreatic resection due to pancreatitis, pancreatic transplantation, or hepatectomy. Pancreatic tumors and pancreatic cancer were excluded from analyses. Exclusion criteria included patients with cholangiohepatitis prior to surgery, bilirubin > 2 mg/dL or more, class B, C on liver function tests, and patients with uncorrected coagulation disorder. Patients whose liver function was expected to be abnormal were excluded. In addition, the liver tissues were confirmed to be normal liver tissues through histology analysis. Donors had an average age of 50.2 ± 16.1; 14 men and 24 women acted as donors. Liver tissues were digested by 0.16% collagenase type I (Worthington Biochemical, NJ, USA) and plated on fibronectin-coated plates (3 μg/cm^2^, Sigma-Aldrich, St. Louis, MO, USA). The cells were cultured in Dulbecco’s modified Eagle’s medium supplemented with 10% fetal bovine serum (FBS), 1% antibiotic-antimycotic, and Glutamax (Life Technologies, Carlsbad, CA, USA). The medium was changed daily during the first 3 days to remove non-adherent cells, and from the fourth day onwards, the medium was changed every 2 or 3 days. Liver cells were passaged when approaching confluence using trypsin-EDTA. After propagation, liver cells from passages 5 to 7 were used for the experiments. To evaluate the growth characteristics of the isolated liver cells, the time required by the cells to proliferate (doubling time) was measured. At every passage, we seeded 2.5 × 10^5^ cells in 100-mm culture dishes. At confluence, the cells were harvested and counted using trypan blue staining. Population doubling time was determined using a previously reported formula [20] and was confirmed using an online doubling time calculator application.

### Characterization of liver cells

We confirmed surface marker expression and differentiation capacity of liver cells. Liver cells at early (1–2), mid (6–7), and late (12–14) passages were blocked with 1% bovine serum albumin and incubated for 1 h at 4 °C with the following anti-human antibodies conjugated with phycoerythrin (PE): mouse IgG1 isotype control, CD31, CD45, CD90, CD73, and CD105 (BD Biosciences, San Jose, CA, USA). Additionally, HLA-DR was analyzed to determine the immune properties. Cells were analyzed using a FACSCalibur device (BD Biosciences). For analyzing albumin expression and ectopic gene expression of PDX1, NEUROD1, and MAFA, liver cells were fixed and permeabilized using eBioscience™ Foxp3/Transcription factor staining buffer set (Thermo Fisher Scientific, Waltham, MA, USA) and were stained using indirect method. Furthermore, the cells were stained using rabbit anti-PDX1, mouse anti-NEUROD1, and rabbit anti-MAFA (1:200; Abcam). For secondary fluorescence labeling, the cells were incubated with anti-rabbit IgG Alexa Fluor 488 and anti-mouse Alexa 555 (1200; Thermo Fisher Scientific). To confirm the introduction of PDX1, NeuroD1, and MAFA genes in cells using flow cytometry, the liver cells in which the transcription factor genes (PDX1, NEUROD1, and MAFA) were not introduced were stained in the same way as gene transduced cells. For albumin control, primary mouse IgG1 isotype control was used as primary antibody instead of mouse anti-albumin.

For adipogenic and osteogenic differentiation, we used the adipogenic or osteogenic differentiation media (Lonza, Basel, Switzerland). After 14 days in culture, the adipogenic culture formed vacuoles. The plates were fixed and stained with Oil Red O (Sigma-Aldrich). The osteogenic differentiation cultures were incubated for 28 days and fixed and stained with 1% Alizarin Red solution pH 4.1 (Sigma-Aldrich). We also confirmed the expression of albumin by isolated liver cells at early (1–2), mid (6–7), and late (12–14) passages. Albumin expression were analyzed by flow cytometry and immunofluorescence staining. For immunofluorescence staining, cells were fixed with 4% paraformaldehyde (Merck, Darmstadt, Germany) and permeabilized with 0.1% Triton X-100. After antibody blocking, primary antibodies were incubated overnight at 4 °C. We used mouse anti-albumin (1:100, Santa Cruz Biotechnology, Dallas, TX, USA) and anti-mouse Alexa 488 (1:200; Thermo Fisher Scientific, Waltham, MA, USA). ProLong Gold antifade reagent with 4′,6-diamidino-2-phenylindole (DAPI, Thermo Fisher Scientific) was used to stain the nuclei and for mounting. The slides were examined using the EVOS® FL auto cell imaging system (Thermo Fisher Scientific).

### Preparation of IPCs and IPC sheets

To induce trans-differentiation, ß cell-related transcription factors were transduced into liver cells. PDX1, NEUROD1, and MAFA were selected and transduced with adenovirus expressing vectors. Ad-CMV-hPDX1, Ad-CMV-hNEUROD1, Ad-CMV-MAFA, and Ad-CMV-GFP were obtained from Vector Biolabs (Burlingame, CA, USA). Ad-RIP-luciferase was generously provided by Orgenesis, Inc. (Germantown, MD, USA). The expression of green fluorescent protein (GFP) after transduction of *GFP* for 2 days was assessed by flow cytometry. Liver cells were transduced with *PDX1* and *NEUROD1* for 2 days, followed by replacement of the medium with medium containing *MAFA* for 3 days. Liver cells were cultured in 10 mM nicotinamide (Sigma-Aldrich), 20 ng/mL epidermal growth factor (PeproTech, Rocky Hill, NJ, USA), and 5 nM exendin-4 (Sigma-Aldrich) included in the culture medium. The optimal MOI was determined according to *GFP* transduction efficiency, cell survival, insulin promotor activity, and insulin gene expression under various conditions. MOIs of the viruses were 500, 250, 50, 200, and 200 for *PDX1*, *NEUROD1*, *MAFA*, *GFP*, and *luciferase*, respectively.

**Fig. 1:**
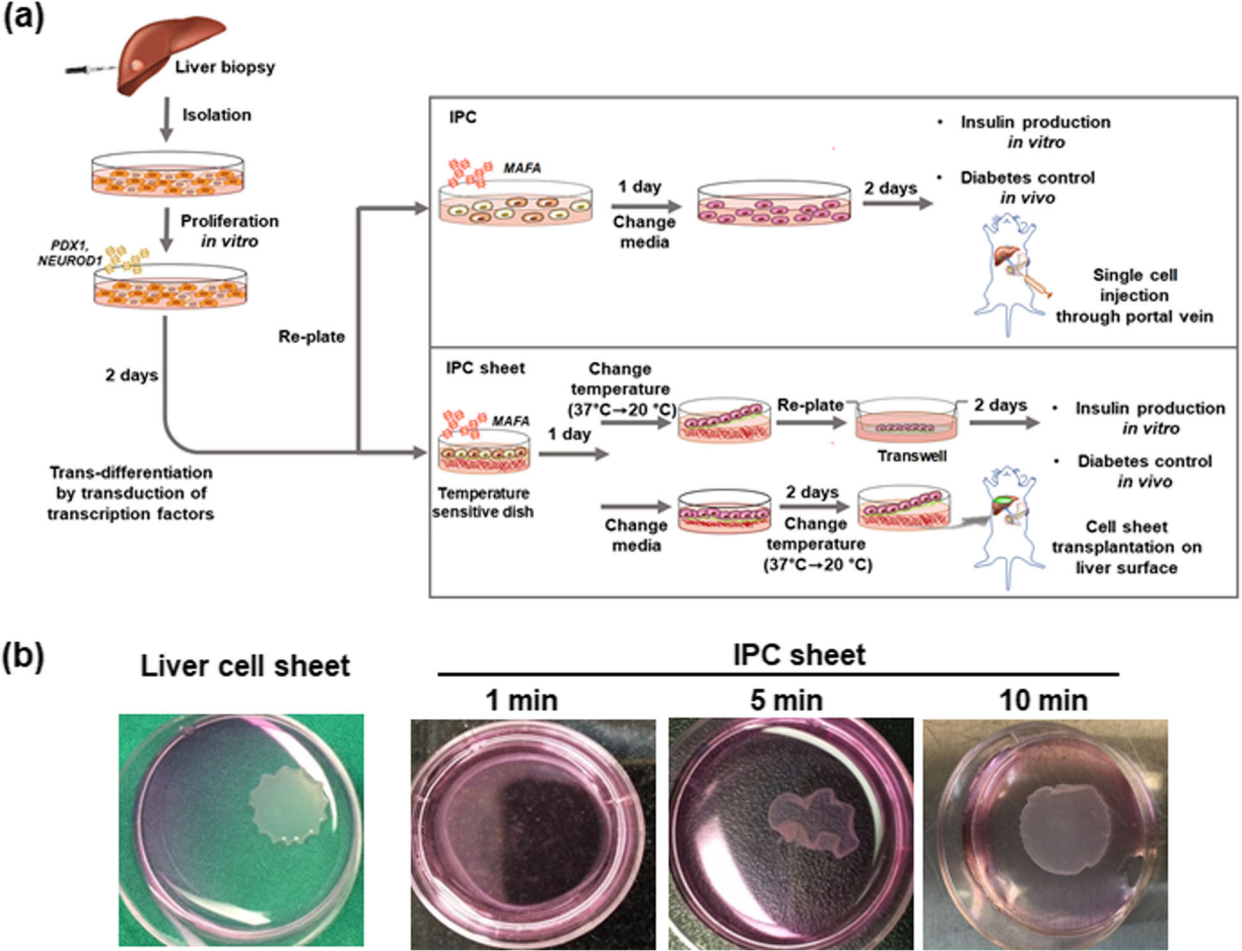
Schematic description of the experimental procedure. **a** Preparation of insulin-producing cells from liver cells using transcription factors, process for the injection of insulin-producing cells (IPCs) through the portal vein, formation of IPC sheets on the temperature-responsive dish, and transplantation of IPC sheets onto the liver surface. **b** Procedure for harvesting of IPC sheets from the temperature-responsive dish. IPC or liver cells were cultured for 2 days and allowed to develop cell-cell interactions. At this point, the temperature was lowered to 25 °C for 10 min, and then, the cell sheet was allowed to float in the dish

The scheme of this study is summarized in Fig. 1a. IPCs and the IPC sheets grown on culture dishes were transduced simultaneously with *PDX1* and *NEUROD1* for 2 days. Next, cells were harvested and re-seeded in commercially available PIPAAm dishes (UpCell; CellSeed, Inc., Tokyo, Japan)—having temperature-responsive properties—to prepare the IPC sheets. For the differentiation of IPCs, cells were re-seeded in culture dishes at a concentration of 10^6^/100 mm. *MAFA* was transduced into IPCs and IPC sheets when cells were re-seeded. On the next day (day 3 after initial virus exposure), the differentiation media were replenished in IPC-seeded culture plates. In the case of IPC sheets, the cell sheets cultured on the UpCell dish were harvested by reducing the temperature from 37 to 20 °C (Fig. 1b) on day 3. For the in vitro assay, the IPC sheet was attached to the insert well with an 8-μm pore size (SPL, Gyeonggi-do, Republic of Korea). Five days after the initial virus exposure, the IPC sheets and IPCs were harvested to analyze mRNA levels and insulin production; these values were compared to those obtained on culturing the IPCs on the monolayer culture dishes.

### Luciferase assay

To optimize the virus transduction conditions, luciferase activity controlled by the insulin promoter was assessed. At day 2, cells were co-infected by adenovirus containing the firefly luciferase gene controlled by the rat insulin promoter (Ad-RIP-luciferase). At day 5 after initial virus exposure, the cells were harvested and lysed in passive lysis buffer (Promega, Madison, WI, USA). Luciferase activity was measured using the Luciferase Assay System (Promega) and normalized to protein levels. Luminescence was detected by a VICTOR2™ 2030 multilabel plate reader (Perkin Elmer, Waltham, MA, USA).

### Real-time quantitative PCR (qPCR)

Total RNA was extracted from liver cells and IPCs on predetermined days using TRIzol reagent (Thermo Fisher Scientific). cDNA was synthesized from 1 μg RNA template by an oligo-dT primer using a SuperScript III First-Strand Synthesis System (Thermo Fisher Scientific). Real-time PCR was performed using LightCycler 480 SYBR Green I Master mix (Roche Applied Science, Mannheim, Germany) in a LightCycler® 480 II real-time thermal cycler (Roche Applied Science). The pancreatic endocrine gene-specific primer sets used are listed in Table 1. Gene expression was normalized to the glyceraldehyde 3-phosphate dehydrogenase housekeeping gene, and relative quantification was performed.

**Table 1:** Primers used for qPCR amplification

Gene		Sequence (5′→3′)	Product size (bp)
Insulin	Forward	GCAGCCTTTGTGAACCAACAC	67
	Reverse	CCCCGCACACTAGGTAGAGA	
Glucagon	Forward	CCCAAGATTTTGTGCAGTGGTT	221
	Reverse	GCGGCCAAGTTCTTCAACAAT	
Somatostatin	Forward	CTGTCTGAACCCAACCAGAC	90
	Reverse	CAGCTCAAGCCTCATTTCAT	
PDX1	Forward	GCATCCCAGGTCTGTCTTCT	140
	Reverse	CACTGCCAGAAAGGTTTGAA	
Ngn3	Forward	GAAAGGACCTGTCTGTCGCT	124
	Reverse	AGGGAGAAGCAGAAGGAACA	
NEUROD1	Forward	CCCTGTACACCCCTACTCCT	92
	Reverse	GAGGCTTAACGTGGAAGACA	
Nkx6.1	Forward	CACACGAGACCCACTTTTTC	76
	Reverse	CCGCCAAGTATTTTGTTTCT	
GAPDH	Forward	GAAGGTGAAGGTCGGAGT	226
	Reverse	GAAGATGGTGATGGGATTTC	

### Immunofluorescence staining

Cells were fixed with 4% paraformaldehyde and permeabilized with 0.1% Triton X-100. After antibody blocking, primary antibodies were incubated overnight at 4 °C. We used rabbit anti-insulin and mouse anti-glucagon (1:1000; Abcam, Cambridge, UK), rabbit anti-PDX1, goat anti-PDX1, mouse anti-NEUROD1, rabbit anti-MAFA (1:200; Abcam), and mouse anti-albumin (1:100, Santa Cruz Biotechnology). For secondary fluorescence labeling, the cells were incubated with anti-rabbit IgG Alexa Fluor 488, anti-rabbit IgG Alex Fluor 555, anti-goat IgG Alex Fluor 488, anti-mouse Alexa 555, and anti-mouse Alexa 488 (1:200; Thermo Fisher Scientific). ProLong Gold antifade reagent containing DAPI (Thermo Fisher Scientific) was used to stain the nuclei and for mounting. The slides were visualized using the EVOS® FL auto cell imaging system (Thermo Fisher Scientific). Under the microscope, the cells with red fluorescence were insulin or glucagon positive and blue fluorescence highlighted the nucleus and counted in 10 randomly selected fields per IPC sheet or IPC cells with total of 3 donors in each group. The results were presented as insulin- or glucagon-positive cells per 100 cells.

### Insulin and C-peptide analysis

To measure the insulin and C-peptide contents in IPCs, cells were washed and lysed with RIPA lysis buffer [21]. Insulin and C-peptide levels were measured using a commercial ultrasensitive insulin ELISA kit (80-INSHUU-E01.1 ALPCO, Salem, NH, USA) and ultrasensitive C-peptide ELISA kit (10-1141-01, Mercodia, Uppsala, Sweden), respectively. Also, insulin secretion was measured in the culture medium of IPCs and IPC sheets by static incubation for 2 days in differentiation culture medium containing 5.5 mM glucose. Insulin and C-peptide contents were ascertained in cells from four different donors. Prior to glucose stimulation of the cells, any residual insulin released from the islets was removed by incubation in serum- and glucose-free RPMI 1640 medium. The tubes were kept at 37 °C for 1 h with shaking, and then, the medium was completely removed by centrifugation and replaced with serum-free RPMI 1640 medium supplemented with 2.8 mM glucose for 1 h. Following collection of the medium, the cells were then incubated with serum-free RPMI 1640 containing 28 mM glucose for an additional 1 h. The supernatant from each sample was collected. The assays were performed in triplicate. The stimulation index was calculated as a ratio between the insulin secreted at high and low glucose media [22].

### Transmission electron microscopy (TEM)

To analyze the granular ultrastructure, the cells were fixed with 1% glutaraldehyde and 1% PFA in 0.1 M sodium cacodylate buffer (pH 7.2) at 4 °C. Specimens were then fixed in 2% osmium tetroxide for 60 min at 4 °C. Dehydration of the fixed samples was performed, and the samples were transferred to Lowicryl resin (Polyscience, Niles, IL, USA). Samples were then sectioned (60 nm) with an ultramicrotome (UltracutUCT, Leica, Wetzlar, Germany) and collected on nickel grids. Post-embedding immunogold labeling was performed for insulin and glucagon labeling using the rabbit anti-insulin (Abcam), mouse anti-glucagon (Abcam), 5-nm colloidal gold conjugated to goat anti-rabbit IgG (Sigma-Aldrich), and 9–11-nm colloidal gold conjugated to goat anti-mouse IgG (Sigma-Aldrich). Following immunogold labeling, the sections were double-stained with 2% uranyl acetate for 20 min and lead citrate for 10 min. The sections were then viewed using TEM.

### Transplantation of IPCs and IPC sheets to diabetic mice

This study was reviewed and approved by the Institutional Animal Care and Use Committee (IACUC No. 2015-12-133) Asan Institute for Life Sciences. The committee abides by the Institute of Laboratory Animal Resources (ILAR) guide. All experiments related to animals were performed in accordance with the relevant guidelines and regulations. To develop insulin-dependent diabetic mouse model, male 8-week-old BALB/c nude mice were treated with 180 mg/kg STZ (Santa Cruz Biotechnology) dissolved in 0.1 M citrate buffer (pH 4.5). After inhalational anesthesia with isoflurane, the abdomen of each mouse was swabbed with Betadine and an approximately 1.5-cm incision was made. Once the portal vein was exposed, it was flat enough to allow a 30-gauge needle to be inserted without restriction. Slight traction was kept on the pancreas near the vein bed with a sterile Q-tip to create tissue tension for injection through the needle. A syringe was attached to the 30-gauge needle, and a suspension of IPCs (1 × 10^6^ cells in 100 μL of phosphate-buffered saline) was released into the portal vein for 1 min. We did not routinely inject more than 10^6^ cells because the injection of greater numbers of cells often was lethal to the mice. Using Q-tips, the injection site was pressed gently for at least 5 min to stop any bleeding. The abdomen was closed and treated with Betadine.

Before cell sheet transplantation, liver capsules at the intended site were removed by swabbing the liver surface. An IPC sheet with CellShifter™ was placed on the liver surface, and the CellShifter™ was removed after 5 min. One IPC sheet contained approximately 10^6^ cells. We transplanted one cell sheet for the biodistribution study to match the cell number with the portal vein injection group. To increase the efficacy of IPC sheets, we transplanted one IPC sheet or three IPC sheets onto the liver of the diabetic mice for efficacy evaluation. Blood glucose was monitored after transplantation using a Codefree blood glucose monitoring system (SD Biosensor, Suwon, Republic of Korea). To confirm human insulin secretion after transplantation, we collected blood serum on the predetermined date and evaluated human serum insulin using an ultrasensitive insulin ELISA kit (80-INSHUU-E01.1 ALPCO, Salem, NH, USA).

### Cell labeling and imaging ex vivo and in vivo

IPCs were labeled with Qdot 800 (Qtracker 800; Molecular Probes, Inc., Eugene, OR, USA). In vivo optical imaging was conducted using an IVIS Spectrum imaging system (Caliper Life Science Inc., Waltham, MA, USA). Images were acquired using an excitation and emission wavelength of 430 and 800 nm, respectively. Fluorescence was quantified as the sum of all detected photon counts per second within a constant region of interest for each transplant site (in vivo). At 7 days after transplantation, the mice were sacrificed and the livers, kidney, spleen, pancreas, and lung were harvested. The fluorescence in each organ was detected (ex vivo image).

### Liver toxicity

Blood samples were collected from the retro-orbital sinus. Hepatic toxicity measurements included ALT and aspartate aminotransferase (AST) in the blood serum at 1, 2, and 5 days after transplantation. Serum ALT and AST levels were analyzed using a model 7180 automatic biochemistry clinical analyzer (Hitachi, Tokyo, Japan).

### Histological analysis

After sacrificing the mice on days 7 and 14, the livers were removed and fixed with 4% formalin. A paraffin block was prepared and cut into 4-μm sections. The tissue slides were deparaffinized and dehydrated. Samples subjected to antigen retrieval were blocked with 3% bovine serum albumin. Immunohistochemistry was performed using primary antibodies for rabbit anti-PDX1 (dilution 1:200, Abcam) and rabbit anti-insulin (dilution 1:1000, Abcam). To confirm neovascularization in transplanted site, we also stained liver tissue using CD31 (dilution 1:1000, Abcam). A DAB Detection Kit (REAL EnVision detection system, DAKO, Glostrup, Denmark) was used for immunohistochemistry. To perform hematoxylin and eosin staining, samples were deparaffinized and dehydrated, followed by staining with hematoxylin (Sigma-Aldrich) and eosin (Sigma-Aldrich).

### Statistical analyses

The data are presented as the mean ± standard deviation, with the number of samples indicated in the figure legends. The statistical significance of the differences between multi-groups was analyzed with one-way ANOVA test and by determining the differences between the means with Tukey’s post hoc test. The statistical significance of the difference between the two groups was analyzed with Student’s *t* test. *P* < 0.05 indicated a statistically significant difference.

## Results

### IPC sheet fabrication

To generative insulin-producing cells, we isolated liver cells from human liver tissues using collagenase. After proliferation, the liver cells were transduced with PDX1 and NEUROD1 for 2 days, followed by replacement of the medium with MAFA-containing medium for 3 days for trans-differentiation. The purpose of this experiment was to compare differentiation in vitro and diabetic control between IPCs and IPC sheets. A schematic summary of the protocol is summarized in Fig. 1a. We simultaneously transduced PDX1 and NEUROD1 for 2 days for both IPCs and the IPC sheets in a conventional culture dish. Then, we harvested cells and re-plated the cells in culture dishes in the presence of MAFA for 3 days. To fabricate the IPC sheets, we harvested and re-plated the cells in temperature-responsive dishes. MAFA was transduced in the same manner as the two-dimensional culture condition. To assess differentiation in vitro, cell sheets were harvested from UpCell dishes by reducing the temperature from 37 to 20 °C (Fig. 1b) at day 3. Each IPC sheet was reattached to the insert well for the in vitro assay. Five days following initial virus exposure, each of the IPC sheet was harvested to analyze mRNA levels and insulin production compared to IPCs cultured on the monolayer culture dish. In case of transplantation, we changed the medium at day 3 and collected IPC sheets for transplantation on the liver surface by reducing the temperature at day 5. We also harvested IPCs from culture dish at day 5 and injected the cells through the portal vein.

### Characterization of human liver cells

**Fig. 2:**
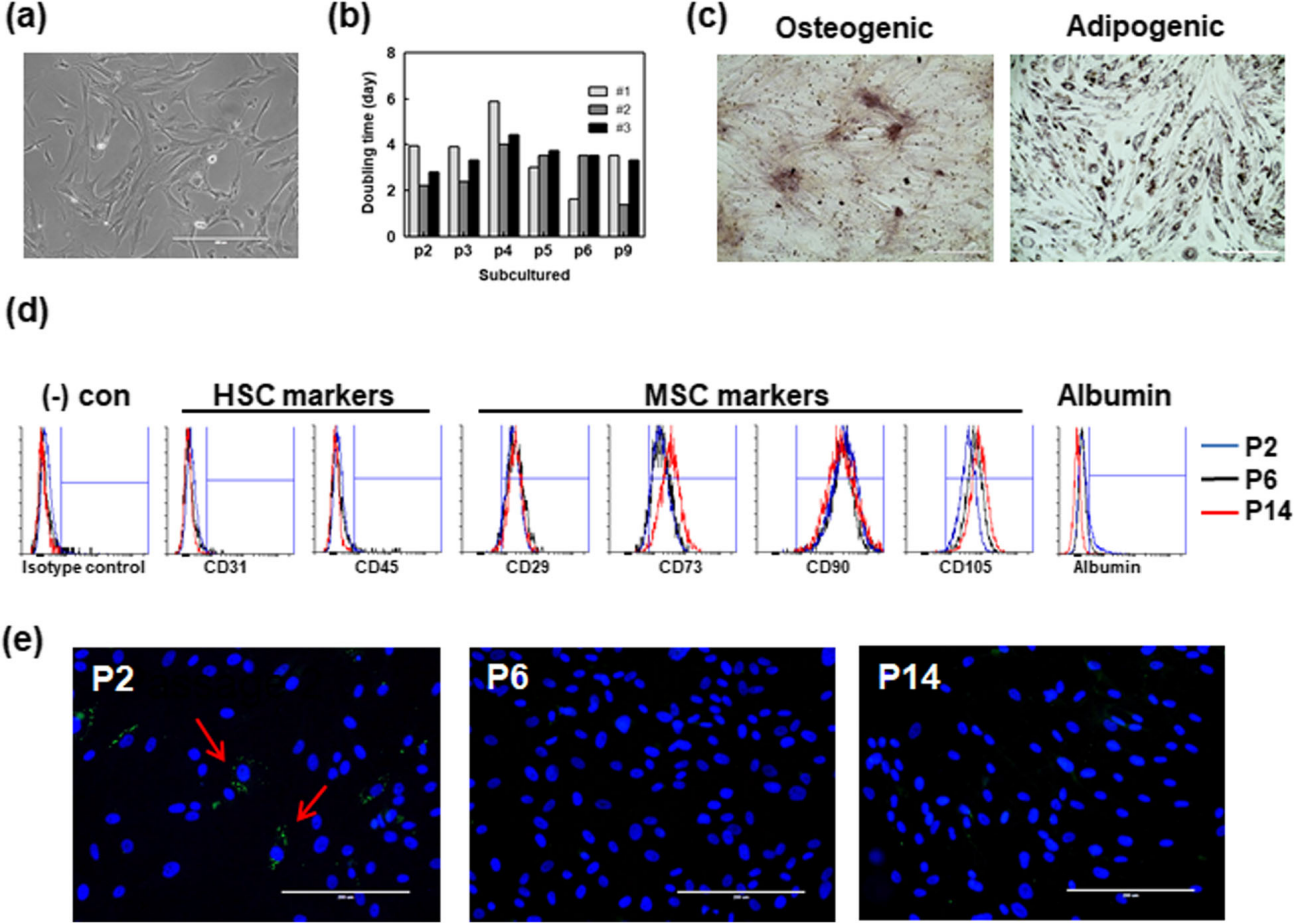
Characteristics of isolated human liver cells and transduction of genes using adenovirus vector. **a** A representative phase contrast morphological image of liver cells from passage 6. **b** Doubling time of liver cells from three different donors in culture media. **c** Differentiation toward the osteogenic (left: Alizarin Red staining) and adipogenic (right: Oil Red O staining) lineages using specific differentiation cocktails for 21 and 14 days in culture, respectively. **d** Expression of surface antigens and albumin on liver cells analyzed by flow cytometry (*n* = 4). Liver cells at early (1–2), mid (6–7), and late (12–14) passages were used. **e** Immunofluorescence staining of albumin in human liver cells at passages 2, 6, and 14. Green, albumin; blue, nucleus; red arrow, albumin-positive cells. Scale bars represent 200 μm

Human liver cells were isolated from liver tissues of 38 donors, including three type 1 diabetes patients. All donors or guardians provided written informed consent for the collection of all samples and subsequent analysis. The donor characteristics of the 38 human livers are provided in the supplement. Mean donor age was 50.2 ± 16.1 years (range 17–79). The primary culture of human liver cells exhibited a heterogeneous phenotype and proliferated efficiently in culture for more than 10 passages. After the 3rd passage, spindle and fibroblast-like cells predominated, while round-shaped cells gradually disappeared (Fig. 2a). Cells proliferated constantly, and a lag time was observed at the 4th or 5th passage (Fig. 2b). Liver cells differentiated into their osteogenic and adipogenic lineages. Osteogenic induction was visualized by Alizarin Red S staining (Fig. 2c, right), and adipogenic differentiation was observed by the presence of lipid droplets detected by Oil Red O staining (Fig. 2c, left). To analyze phenotype, flow cytometry was performed on isolated liver cells at early (passage 1–2), mid (passage 6–7), and late (passage 12–13) passages (Fig. 2d and Supplementary Table 2). Cells isolated from human liver tissues were positive for CD29, CD90, and CD105, whereas they were negative for CD31 and CD45 at all passages. The prevalence of CD73-positive cells exceeded 90% at all time points and increased with continued passaging. Cells were also negative for human leukocyte antigen–DR isotype (HLA-DR). We also confirmed the expression of hepatic markers in liver cells by flow cytometry and immunocytochemistry. Albumin was partially expressed at the initial stage (19.8 ± 10.5), but isolated liver cells in culture displayed decreased albumin expression at late passages (0.5 ± 0.2).

### Expression of ectopic transcription factors using adenoviral vectors

**Fig. 3:**
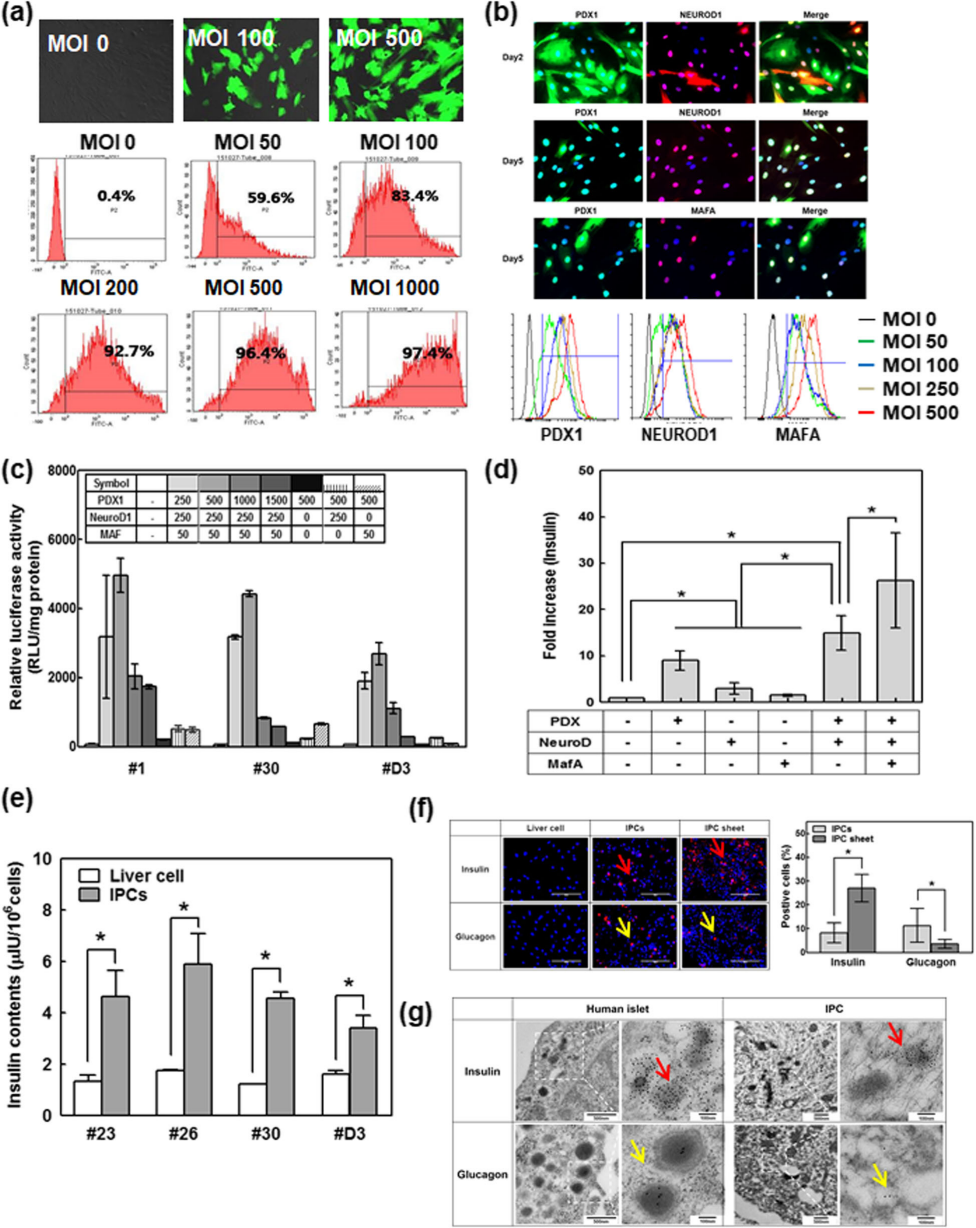
Ectopic co-expression of transcription factors in the liver in vitro promotes endocrine differentiation. **a** GFP expression in liver cells (passage 6) after Ad-CMV-GFP transduction for 2 days with various MOIs analyzed by flow cytometry and fluorescence imaging (*n* = 3). Scale bars represent 400 μm. **b** Multiple immunofluorescence staining of human liver cells. Human liver cells (passage 6) were simultaneously treated with Ad-CMV-PDX1 (500 MOI) and Ad-CMV-NEUROD1 (250 MOI) for 2 days and then re-plated with Ad-CMV-hPDX1 (MOI 50) for 3 days. Green, PDX1 or MAFA; red, NEUROD1; blue, nucleus. Scale bars represent 200 μm. Flow cytometry data of PDX1, NEUROD1, and MAFA transduced liver cells with various MOIs (lower panel). **c** Cultures were co-infected with the combined transcription factors and with adenovirus carrying a luciferase reporter gene under control of the insulin promoter (Ad-RIP-luciferase: 200 MOI), and luciferase activity was measured. The results are expressed as relative light units (RLU)/mg protein (*n* = 3). **d** Real-time qPCR analysis of insulin gene expression in liver cells, insulin-producing cells (IPCs) treated with various combinations of transcription factors and human pancreatic islets (*n* = 3). The results normalized to the GAPDH gene expression for the same cDNA sample are represented as the relative levels of the mean ± SD; *P* < 0.05 indicates a significant difference. **e** Production of insulin by IPCs. Each data point represents the mean ± SD in cells isolated from four different donors compared to that in control virus-treated cells. *P* < 0.05 compared to liver. **f** Immunocytochemistry of insulin and glucagon in the liver cell, IPCs, and IPC sheet. Representative insulin and glucagon staining using immunocytochemistry (left). Red, insulin or glucagon; blue, nuclei. Scale bars represent 200 μm. Percentage of insulin- or glucagon-positive cells in IPCs and IPC sheet (right). **g** TEM images of IPCs. The red arrow indicates an insulin immunogold aggregate (9–11 nm), and the white arrow indicates glucagon immunogold aggregate dot (5 nm)

To determine the optimal dose for the transduction of liver cells using adenoviral vectors, liver cells at passage 6 from three different donors were infected with adenovirus expressing green fluorescent protein under the control of a cytomegalovirus promoter (Ad-CMV-GFP) at various multiplicities of infections (MOIs) from 50 to 1000. Flow cytometry analysis was performed 2 days after infection to determine the number of GFP-positive cells. At MOI 50, more than 50% of cells were transduced with Ad-CMV-GFP (Fig. 3a). The intensity of GFP fluorescence was dose-dependently increased, and the number of GFP-positive cells at MOI 200 was greater than 90%. Immunocytochemistry confirmed ectopic gene expressions of PDX1, NEUROD1, and MAFA in the nuclei of liver cells using various MOIs (Figure S1) for 2 days. At MOIs of 250, 500, and 1000, over 90% of cells were positive for PDX1, NEUROD1, and MAFA, respectively. Also, we confirmed co-expressions of PDX1, NEUROD1, and MAFA in nuclei after liver cells were transduced with Ad-CMV-hPDX1 (MOI 500) and Ad-hNEUROD1 (MOI 250) for 2 days and consequently with Ad-CMV-MAFA (MOI 50) for 3 days. PDX1 and NEUROD1 were co-expressed in most cells, while MAFA was partially expressed (Fig. 3b).

The cooperative action of various transcription factors is required for efficient trans-differentiation of adult cells to IPCs. The MOI of each factor was titrated to result in maximal insulin promoter activity, which was determined by luciferase assay and insulin gene expression analysis, with minimal adverse effects on gene-infected cell viability. To determine the optimal dose for the transduction of liver cells using adenoviral vectors, liver cells at passage 6 were infected with Ad-CMV-hPDX1 and Ad-hNEUROD1 at various MOIs for 2 days and consequently with Ad-CMV-MAFA at MOI 50 for 3 days. As the MOI of *PDX1* increased, luciferase activity also increased (Fig. 3c). However, an MOI of *PDX1* greater than 500 resulted in high rates of cell death. Combined treatment of the three different transcription factors produced the highest luciferase activity compared with that in cells treated with *PDX1* alone or with *PDX1*/*NEUROD1*. Luciferase activity assessed in liver cells from three different donors showed a similar pattern. Also, we confirmed insulin gene expression after transduction with ectopic genes. The combination of PDX1/NEUROD1 and MAFA transcription factors increased the intensity of insulin mRNA levels compared with the levels in cells treated individually with PDX1, NEUROD1, or MAFA, or with PDX1/NEUROD1. Insulin gene expression was substantially increased (26-fold) in adult human liver cells co-infected with the three recombinant adenoviruses compared with the expression in non-treated liver cells (Fig. 3d). Based on these results, the MOIs of *PDX1*, *NEUROD1*, and *MAFA* were optimized to 500, 250, and 50, respectively.

### Confirmation of the trans-differentiation of liver cells to IPCs

Insulin production was assessed by measuring insulin contents in IPCs and in undifferentiated liver cells. As shown in Fig. 3e, the insulin content in IPCs was significantly increased in four different donors compared to the content in liver cells. Particularly, liver cells from donor number D3 were isolated from a type 1 diabetes patient. To confirm the trans-differentiation of liver cells to IPCs, immunohistochemistry and insulin contents were assessed by incubation of adult liver cells for 5 days after initial exposure to viral treatment. Compared to IPCs, insulin-positive cells were identified in the IPC sheets at a higher rate, whereas glucagon-positive cells were more prominent in IPCs (Fig. 3f). But to determine the granular protein composition, immunogold labeling of insulin and glucagon in IPCs was followed by transmission electron microscopy (TEM) examination (Fig. 3g). Insulin protein aggregates were observed in IPCs, while glucagon protein was spread in the cytosol in very small amounts. While we confirmed insulin expression in IPCs by TEM and insulin production in an ELISA assay, the insulin level was much lower than that in natural human islets.

### Cell sheet formation enhances the differentiation function of IPC

**Fig. 4:**
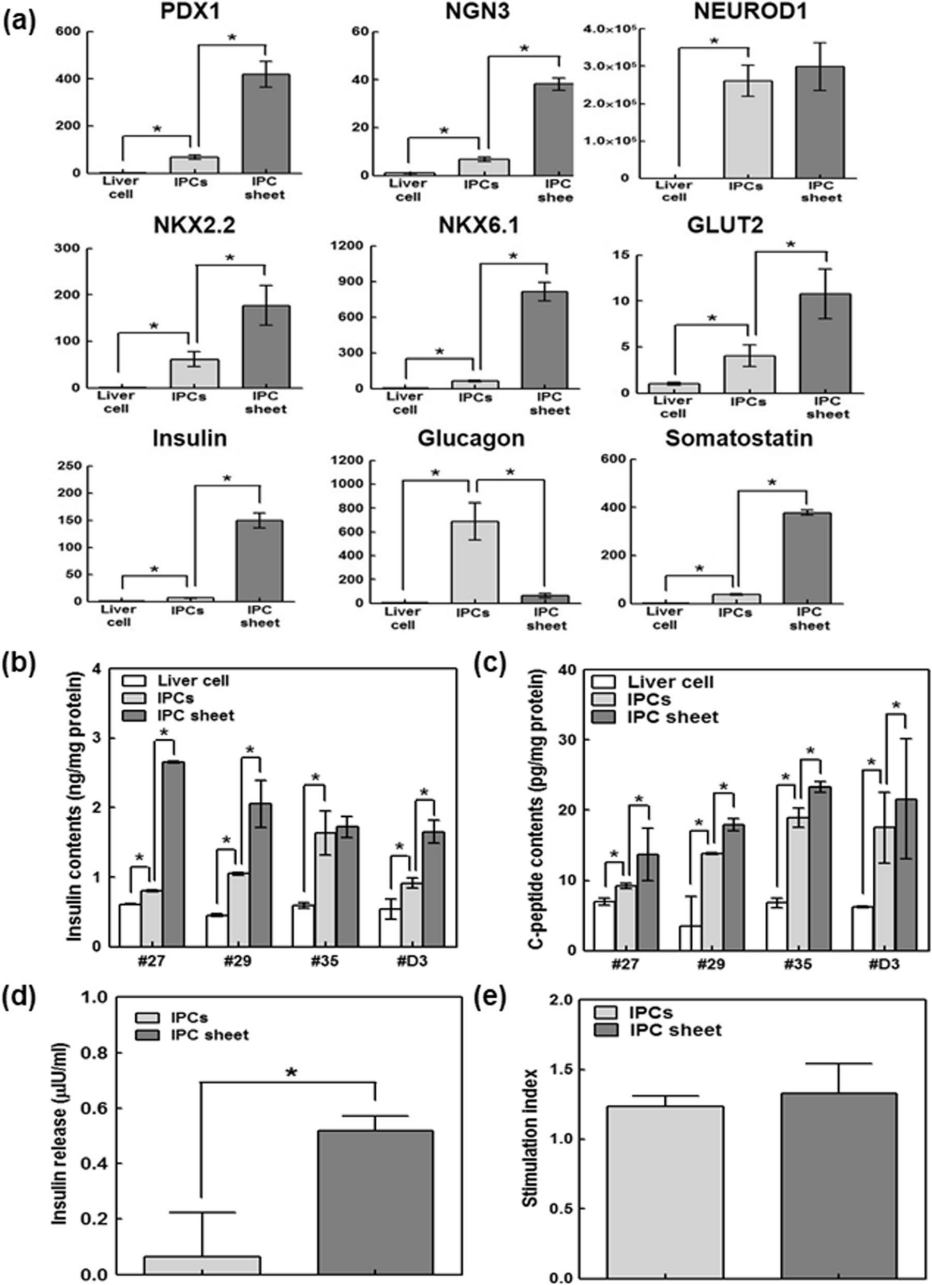
Comparison of mRNA expression and insulin production between insulin-producing cell (IPC) sheets and IPCs cultured on a conventional cell culture plate. **a** Real-time qPCR analysis of gene expression in liver cells and IPCs. IPCs were confirmed to express endocrine hormones, including insulin, glucagon, somatostatin, and pancreatic-specific genes, including *PDX1*, *NGN3*, *NEUROD1*, *NKX6.1*, and glucose transporter 2, compared to those in liver cells. The results normalized to GAPDH gene expression for the same cDNA sample are represented as the relative levels of the mean ± SD; *P* < 0.05 indicates a significant difference. **b** Insulin and **c** C-peptide contents and **d** insulin secretion in IPCs and IPC sheets after 5 days of culture. **e** Glucose stimulated insulin secretion index. The stimulation index was calculated as a ratio between the insulin secreted at high and low glucose media

To compare the trans-differentiation of IPCs under different culture conditions, the mRNA levels of endocrine hormone and pancreatic transcription factors were determined after 5 days of culture. As shown in Fig. 4a, IPCs expressed endocrine hormones, including insulin, glucagon, and somatostatin, and pancreatic-specific genes, including *PDX1*, *NEUROD1*, *MAFA*, *NGN3*, *NKX2.2*, *NKX6.1*, and glucose transporter 2 (*GLUT2*) compared to IPCs cultured on conventional culture plates. In particular, the insulin mRNA level of IPCs cultured with the sheet structure was significantly higher than that of IPCs, whereas IPCs in the sheet structure did not have detectable levels of glucagon mRNA. Similarly, pancreatic transcription factors related to beta cell trans-differentiation were higher in IPC sheets than in single cell cultures. This finding suggested that sheet formation can induce trans-differentiated liver cells in the beta cell lineage but represses unwanted trans-differentiation into the alpha cell lineage. Additionally, insulin and C-peptide production and insulin secretion were significantly increased in IPC sheets compared to IPCs (Fig. 4b, c). To further examine physiological insulin secretion, we evaluated the level of insulin secretion at two different glucose concentrations (2.5 mM and 25 mM). Although the secretion in IPC sheets tended to be slightly higher than that in IPCs, the stimulation index (insulin release at high glucose/low glucose) was not significantly different between IPCs and IPC sheets (Fig. 4e).

### Transplantation of IPC sheet on liver surface and IPCs through the portal vein

**Fig. 5:**
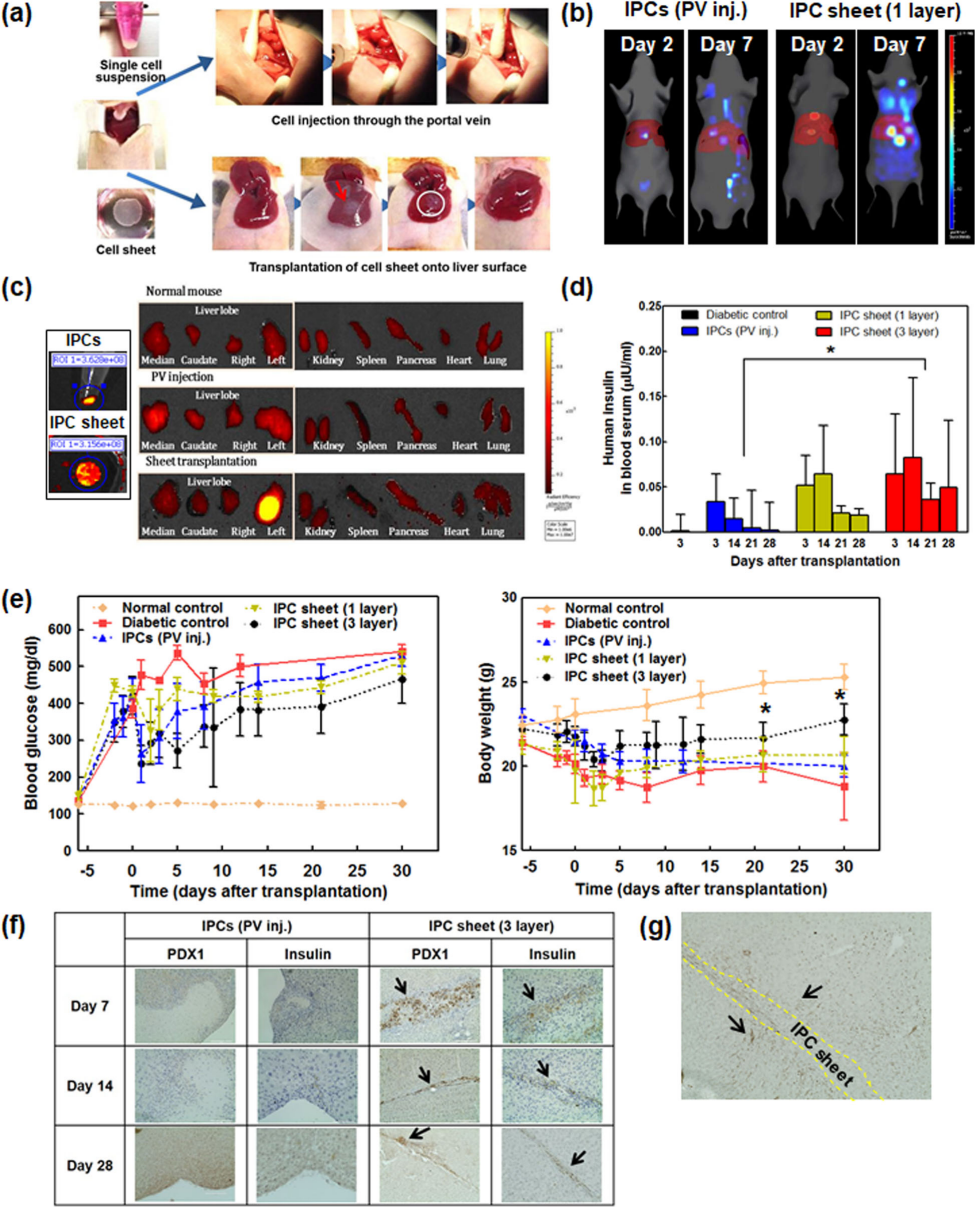
Comparison of transplantation efficiency between transplantation of insulin-producing cell (IPC) sheets (one or three sheets) on the liver surface and injection of IPCs through the portal vein. **a** Photographs of experimental protocols of IPC portal vein injection (upper) and IPC sheet transplantation on the liver surface of immune-deficient diabetic mouse. **b** Distribution of transplanted or injected IPCs in mice. The distribution of transplanted IPCs in recipient mice was examined with an in vivo imaging system. Optical imaging of IPC labeled Qdot 800 in vivo and ex vivo. Fluorescence color images were processed using Living Image V.3.2. A grayscale body image was collected and overlaid by a pseudo-color image representing the spatial distribution of the detected photons. **c** Fluorescence images of IPCs and IPC sheet before transplantation (left). Seven days after treatment, the liver, kidney, spleen, pancreas, heart, and lung were harvested, and ex vivo fluorescent images were acquired (right). **d** Human insulin levels in blood serum from mice transplanted with one or three cell sheets and PV injected. Diabetic mice without transplants were negative controls. *N* = 4 or 5. The asterisk symbol (*) indicates a significant increase in the insulin level of IPC sheets (three layers) upon IPC injection at each time point; *P* < 0.05. **e** Therapeutic effects of insulin-producing cell (IPC) sheet transplantation in diabetic nude mice. Two weeks before transplantation, mice were induced with insulin-dependent diabetes using streptozotocin (180 mg/mL). At day 0, diabetic nude mice were transplanted with three IPC sheets or injected with 10^6^ IPCs through the portal vein. Diabetic nude mice and wild-type mice were used as negative and positive controls, respectively. At different time points, non-fasting blood glucose levels and body weights were measured in sheet transplanted on liver surface, portal vein injected IPCs, and diabetic control (*n* = 5). The asterisk (*) indicates a significant increase in body weight upon IPCs; *P* < 0.05. **f** Immunohistochemical analyses of PDX1and insulin in the liver at 7, 14, and 28 days after transplantation of IPCs and IPC sheets. Arrows indicate positive cell staining. Original magnifications are × 200 (PDX1) and × 400 (insulin). **g** Immunohistochemical analyses of CD31 in the liver at 28 days after transplantation of IPC sheets. Arrow indicated positive cell staining. Original magnifications are × 200

The drug STZ is known to induce diabetes. For the mouse model of diabetes, STZ (180 mg/kg) was administered by single and intraperitoneal injection. When blood glucose levels were ≥ 300 mg/dL for more than 1 week, mice were either transplanted with an IPC sheet onto the liver surface or injected with IPCs through the portal vein. Figure 5a illustrates the process for IPC intraportal injection (upper) and cell sheet transplantation onto the liver surface (lower) in the animal study. A single cell sheet (10^6^ cells) was transplanted onto the liver surface to transplant the same number of cells as were administered by intraportal injection. Five of 25 mice injected 10^6^ IPCs died during portal vein injection or from postoperative complications, while all mice survived sheet transplantation.

To track transplanted cells, cells were labeled with Qdot 800, which supports intense fluorescence under various biological conditions and less auto-fluorescence in tissue [23]. The fluorescent IPC signal was detected in the region of the liver in transplanted mice at days 2 and 7, while a weak fluorescent signal was detected in the intraportal injection group on day 2. Mice were sacrificed, and internal organs were imaged ex vivo at 7 days post-transplantation. The greatest fluorescent signal was detected in the liver of the sheet-transplanted group (Fig. 5b, c).

We examined the therapeutic effects of IPC sheets in comparison to IPC intraportal injection for diabetes in nude mice. To confirm human insulin secretion after transplantation, human insulin levels were evaluated on days 3, 14, 21, and 28 (Fig. 5d). The mice transplanted three IPC sheets showed higher insulin levels than IPC-injected mice. After transplantation, a decrease in blood glucose levels from diabetic mice was observed at first week in all IPC and IPC sheet groups. Although an increase in blood glucose levels was observed in both the IPC injection and cell sheet groups, the mice transplanted three IPC sheets showed the lowest blood glucose levels during the observation period. Additionally, mice transplanted with the one or three IPC sheets showed an increase in body weight, while the single cell injection and diabetic control groups showed decreased body weights (Fig. 5e). After 1, 2, and 4 weeks of transplantation, PDX1 and insulin-expressing cells were detected by immunohistochemistry staining on the liver surface only in the IPC sheet group (Fig. 5f). Also, we confirmed CD31-positive cells to detect neovascularization in the transplantation site. Abundant blood vessels were shown around the transplanted cell sheet, and some CD31-positive cells were also found in the IPC sheets (Fig. 5g).

### Liver toxicity after transplantation and histology

**Fig. 6:**
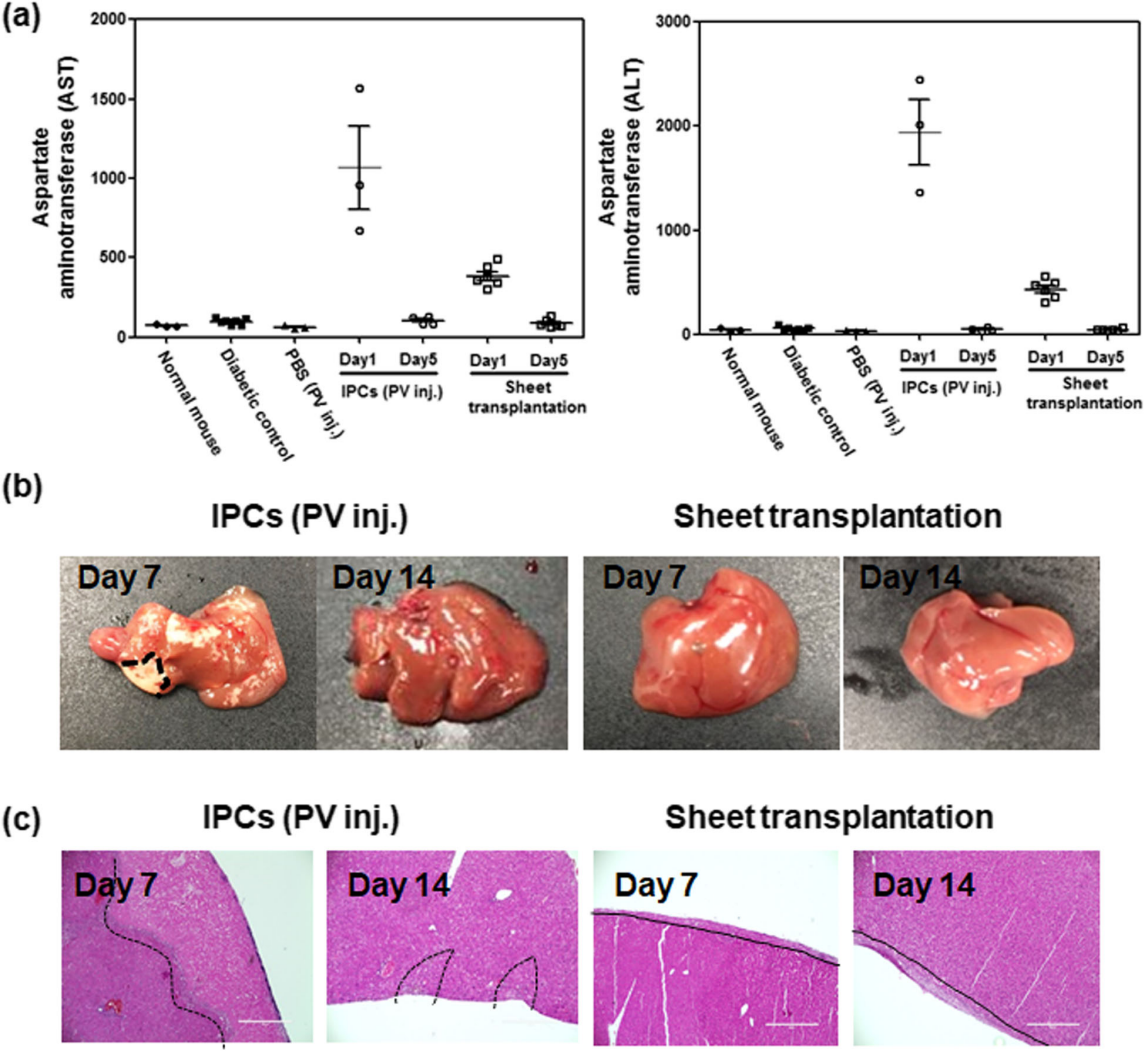
**a** Levels of liver toxicity. Normal mice, diabetic controls, and mice injected with phosphate-buffered saline via the portal vein did not show increased serum aspartate aminotransferase (AST) and alkaline aminotransferase (ALT). AST and ALT levels rapidly increased after injection of insulin-producing cells (IPCs) and transplantation of IPC sheets at day 1 and recovered to baseline levels after 5 days. AST and ALT levels of mice injected with IPCs were higher than in mice transplanted with IPC sheets. **b** One and 2 weeks after IPC sheet transplantation or IPC injection, the liver was observed macroscopically. The dotted line indicates the necrotic area. **c** One and 2 weeks after transplantation, the livers of both groups were analyzed by hematoxylin and eosin staining. The dotted line indicates the necrotic area, and solid line indicates the sheet-transplanted area on the liver surface

Liver function and histological examinations were performed to evaluate liver toxicity after intraportal injection or cell sheet transplantation of IPC. One day after cell injection through the portal vein, ALT and AST increased rapidly and then recovered gradually (Fig. 6a). In the IPC sheet group, ALT and AST levels were as low as one third of the value in the injection group. At 7 and 14 days after transplantation, the liver surface of transplanted animals was visually observed (Fig. 6b). In the PV injection group, white necrotic tissue was observed in many parts of the liver margin. Although this was completely recovered after 2 weeks, a morphological change was also observed. In contrast, these necrotic tissues or morphological changes were not observed in the IPC sheet group. Histological evaluation showed similar results (Fig. 6c). In the injection group, the necrosis structure was confirmed. No necrotic region was observed in the sheet group, and the sheet adhered well to the liver surface.

## Discussion

Transplantation of autologous insulin-producing cells (IPCs) may be an ideal diabetic treatment for overcoming the limitations of current pancreas and islet transplantation, including the shortage of donors, immune reactions of the allograft, and low graft survival [24]. Various techniques and an alternative cell source have been actively reported for the development of new IPCs using differentiation from stem cells [25, 26], the proliferation of existing adult β cells [27], and reprogramming of adult somatic cells [28, 29]. A recent report showed that cells isolated from the liver have high differentiation potential for the pancreatic lineage because the liver has the same origin as the pancreas.

In this study, we isolated and used liver cells according to the previous reported method in Sarah et al. Adherent cells isolated from the liver acquired mesenchymal-like characteristics and considerable cellular plasticity, as has previously been reported [9]. According to the results of genetic lineage-tracing of liver cells, cells isolated from the liver were partially positive for hepatic markers such as albumin and AFT and simultaneously positive for MSC markers. However, they were negative for hematopoietic (CD45, CD31) and hepatic progenitor markers (CK19, EpCAM) [9]. Authors said that the isolated liver cells can proliferate and obtain mesenchymal characteristics and cellular plasticity through EMT in in vitro cultures. Similarly, in this study, albumin-positive cells were identified through immunocytochemistry staining and flow cytometry at the initial stage of isolation, and it was also confirmed that they were positive for MSC surface markers and possessed differentiation ability.

Numerous studies have been conducted in which differentiation of insulin-producing cells was effected by inducing the expression of transcription factors such as PDX1, NGN3, MAFA, and PAX4, which play important roles in pancreatic development [30, 31]. Recently, Ferber et al. showed that mature β cell-like characteristics were induced when three pancreatic transcription factors, PDX1, PAX4, and MAFA, were sequentially supplemented in a direct hierarchical manner [14]. It is important that transcription factors are turned on and off at appropriate times for determining cell fate. PDX1 alone has little activity or it induces other pancreatic cells; however, it becomes a potent factor when it interacts with NEUROD1 [32, 33]. Another important transcription factor for β cell-specific differentiation is MAFA [34–37]. Thus, we used PDX1, NEUROD1, and MAFA to induce IPC from liver cells. Based on these previous studies, PDX1 was essentially used to induce trans-differentiation from mesoderm to endoderm. In addition, NEUROD1, which can simultaneously induce endoderm while enhancing the effect of PDX1 by binding to the same site as PDX1, was simultaneously added. In particular, as the switch from MAFB to MAFA expression is essential for maturation of beta cells, *MAFA* was introduced 2 days after the start of differentiation to improve insulin production through maturation of IPC. *PDX1* at an MOI 500 was used as a key factor because PDX1 plays a crucial role in pancreatic organogenesis and beta cell function. The MOI of *NEUROD1* and *MAFA* was optimized based on insulin promoter activity, insulin gene expression, and minimal adverse effects on infected cell viability. This combination increased the intensity of insulin promoter activity and mRNA level of insulin genes compared to that in cells treated with either PDX1 or NEUROD1, MAFA alone. In this study, *PDX1* and *NEUROD1* were added at the same time, and *MAFA* was sequentially induced 2 days later to maximize trans-differentiated cell maturation, as indicated by increased insulin gene expression. Insulin protein was confirmed to be expressed by immunohistochemistry, TEM image, and ELISA. Unlike fully mature beta cells, granule formation was not complete, but some aggregates of insulin protein were identified in TEM image. However, glucagon did not form an aggregate and was distributed in the cytosol to a small degree.

It is known that cell-cell interaction of islets is important for insulin production and secretion [38, 39]. In our study, when cell sheets were formed, insulin gene expression and protein production were significantly increased. Also, several transcription factors were significantly increased in the cell sheet group. Interestingly, the expression of glucagon was dramatically reduced in the cell sheet group. In previous reports, Nkx6.1 reduces the expression of the glucagon gene, which explains why these transcription factors in the cell sheet group decreased glucagon expression and thus increased the purity of insulin-producing cell differentiation [40]. Insulin and C-peptide production and insulin secretion also significantly increased in the sheet group. Although the cell sheet’s ability to induce differentiation in IPC was confirmed in this study, further studies are required to determine the mechanisms underlying cell sheet formation and IPC differentiation and maturation.

A cell sheet-based tissue engineering approach has attracted attention in cell transplantation therapy because of its potential to deliver a large number of cells to the desired organ without surgical procedures [15, 41, 42]. Cell sheets maintain adhesive protein layers that facilitate attachment to other tissues. The cell sheet technique also makes the delivery of target molecule safe. In the successful clinical trial of intraportal islet transplantation by Shapiro et al., islet transplantation was exclusively conducted via the portal vein [17, 43, 44]. When the islet is administered directly through the portal vein, many cells engraft in the liver through smaller venues, and the liver can supply sufficient blood in a near-physiological insulin delivery environment. However, several concerns remain regarding intraportal islet infusion, including procedure-related complications, bleeding, hepatic hypertension, thrombosis, and instant blood-mediated inflammatory reaction [16]. Various alternative sites for successful islet engraftment have been proposed for the islet or IPC, including the kidney capsule, omentum, skin, and cornea. Subcutaneous engraftment has also been proposed. Recently, Alejandro et al. reported that a patient who underwent islet transplantation on the omentum showed stable glycemic control without exogenous insulin and episodes of hypoglycemia at 12 months [45]. Although some locations may be advantageous in experimental models, their feasibility and translation into clinical settings are limited [46]. An ideal transplantation site should include a sufficient blood supply without any surgical risk. The liver is ideal for cell transplantation because of the high blood supply, near-physiologic insulin delivery environment, and long clinical application history. If the IPCs’ construct can be directly transplanted onto the liver surface, rather than via portal vein injection, this may be an ideal transplantation technique for ensuring safety and increasing survival and near-physiological insulin delivery. In this study, we confirmed that the IPC sheet improved the delivery efficacy of IPCs in the liver and decreased the risk of PV injection. Additionally, IPC sheet formation may enhance the differentiation function of IPCs in vitro by maintaining cell-cell interactions. To identify the potential of clinical applications for IPC sheet transplantation, we assessed not only glycemic control efficiency but also operative feasibility, biodistribution, and liver toxicity, compared to the IPC intraportal injection technique. To develop an insulin-dependent diabetic mouse model, nude mice were treated with 180 mg/kg streptozotocin once. In this study, cells were isolated from human liver tissues and transduced with the genes for human transcription factors PDX1, NEUROD1, and MAFA to induce their differentiation into IPCs. Mice cells could not be used because we have not evaluated the availability of human transcription factors in mouse cells in any previous study. Therefore, athymic nude mice were used as the diabetic animal model to control immunity for human cell transplantation.

There are a million cells per IPC sheet, which has the same cell count for the IPC PV injection group. Although cell sheet formation enhanced the differentiation of IPCs in vitro, there were no significant differences in vivo between the mice injected with 10^6^ IPCs and those transplanted with the IPC sheet. This is because the number of differentiated cells is insufficient for regulating blood glucose levels when a single cell sheet is used. However, increasing the number of IPC sheets (three layers) could decrease the glucose levels and increase the body weight. The same has been confirmed in case of human insulin secretion in blood. Mice transplanted with three IPC sheets showed higher insulin levels than did IPC-injected mice. It is important to improve the differentiation efficacy of IPCs, and the delivery of large quantities of cells to the target site in a non-toxic manner can significantly improve their function. Although sheet formation did not completely regulate blood glucose levels when the same number of cells was used, the method was advantageous as the cell number could be increased if necessary. It is difficult to introduce more than a certain number of cells through portal vein injection, which is the method used in clinical practice, owing to the risks of serious side effects, such as embolisms. Also, immunohistochemical analyses showed that IPC sheet-transplanted livers stained positively for PDX1 and insulin at 1, 2, and 4 weeks post-transplantation.

After injecting the IPC suspension via the portal vein, and transplantation of the IPC sheet onto the liver of diabetic nude mice, cell biodistribution was assessed by in vivo/ex vivo fluorescence image tagging Qdot. The IPCs were detected in the liver in the cell sheet-transplanted region of mice at days 2 and 7, while the intraportal injection group showed only a weak signal at day 2. This was confirmed by ex vivo fluorescence image and histology.

Increasing transplantation efficiency and reducing toxicity are important factors for clinical application. When we injected 10^6^ differentiated IPCs through the portal vein, 80% of animals survived. In the preliminary study, mice transplanted with 1.5 × 10^6^ and 2 × 10^6^ IPCs died at rates of 40% and 90% during surgery, respectively (data not shown). In this study, when high doses of cells (1.5 × 10^6^ and 2 × 10^6^) were administered through the portal vein, most animal deaths occurred in the middle of surgery or within tens of minutes of surgery. In general, portal vein thrombosis rates in humans and thromboembolisms and subsequent mortality in murine models are reported to occur due to injection through the portal vein [47]. Death of high-dose cells through PV injection is caused by thromboembolisms formed by excess cells. However, no mice transplanted with 1, 3, or 5 layers of the IPC sheet died as a consequence of the operation. Mice injected with the IPCs through the portal vein showed liver toxicity and histologic damage to many parts of the liver margin. However, the IPC sheet showed no significant liver toxicity in our study. These results showed that directly transplanting the IPC sheet onto the liver surface is expected to significantly lower the risks related to current intra-portal vein injection, which include thrombosis, bleeding, and liver toxicity.

In conclusion, cell sheet formation enhanced the differentiation function and maturation of IPC in vitro. Additionally, parameters for clinical application such as distribution, therapeutic efficacy, and toxicity were favorable. The cell sheet technique may be used with IPCs derived from various cell sources in clinical applications.

## Supplementary Information

**Additional file 1.**

## Data Availability

The data that support the findings of this study are available on request from the corresponding author.

## Abbreviations

Ad-CMV-GFP: Adenovirus with CMV promoter-driven expression of GFP
Ad-CMV-MAFA: Adenovirus with CMV promoter-driven expression of MAFA
Ad-CMV-hNEUROD1: Adenovirus with CMV promoter-driven expression of NEUROD1
Ad-CMV-PDX1: Adenovirus with CMV promoter-driven expression of PDX1
Ad-RIP-luciferase: Adenovirus with rat insulin promoter-driven expression of luciferase
ALT: Alanine transferase
AST: Aspartate transaminase
GFP: Green fluorescent protein
GLUT2: Glucose transporter 2
IPC sheet: Insulin-producing cell sheet
IPCs: Insulin-producing cells
MAFA: v-maf musculoaponeurotic fibrosarcoma oncogene family, protein A
MOI: Multiplicity of infection
NEUROD1: Neuronal differentiation 1
NGN3: Neurogenin-3
NKX2.2: NK2 homeobox 2
NKX6.1: NK6 homeobox 1
PAX4: Paired box 4
PDX1: Pancreatic and duodenal homeobox 1
PIPAAm: Poly(*N*-isopropylacrylamide)
PV: Portal vein
RIP: Rat insulin promotor
STZ: Streptozotocin
TEM: Transmission electron microscopy
