# Supplementary material for: Improvement of the therapeutic capacity of insulin-producing cells trans-differentiated from human liver cells using engineered cell sheet
Source: Stem Cell Res Ther. 2021 Jan 6;12:3. doi: 10.1186/s13287-020-02080-0 (PMC7786992; doi:10.1186/s13287-020-02080-0)

Supplementary Information

**Improvement of the therapeutic capacity of insulin-producing cells trans-differentiated from human liver cells using engineered cell sheet**

Yu Na Lee^1, +^, Hye-Jin Yi^1, +^, Eun Hye Seo^1^, Jooyun Oh^1^, Song Lee^1^, Sarah Ferber^2^, Teruo Okano^3, 4^, In Kyong Shim^1,^ *,Song Cheol Kim^1, 5,^ *

^1^ Asan Institute for Life Sciences, Asan Medical Center, University of Ulsan College of Medicine, Seoul, Korea

^2^ Sheba Regenerative Medicine, Stem Cells and Tissue Engineering Center, Sheba Medical Center, Tel-Hashomer, Israel

^3^ Institute of Advanced Biomedical Engineering and Science, Tokyo Women's Medical University, Tokyo, Japan

^4^ Cell Sheet Tissue Engineering Center, Department of Pharmaceutics and Pharmaceutical Chemistry, University of Utah, Salt Lake City, USA

^5^ Department of Surgery, Asan Medical Center, University of Ulsan College of Medicine, Seoul, Korea

Table S1. Characteristics of donors from whom liver cells were isolated

| Donor | Age | Sex |
| --- | --- | --- |
| #1 | 66 | F |
| #2 | 61 | M |
| #3 | 50 | M |
| #4 | 69 | F |
| #5 | 53 | M |
| #6 | 69 | M |
| #7 | 54 | F |
| #8 | 67 | M |
| #9 | 53 | F |
| #10 | 53 | M |
| #11 | 42 | M |
| #12 | 74 | M |
| #13 | 79 | F |
| #14 | 71 | M |
| #15 | 43 | M |
| #16 | 32 | F |
| #17 | 39 | M |
| #18 | 77 | F |
| #19 | 36 | F |
| #20 | 34 | F |
| #21 | 55 | F |
| #22 | 31 | F |
| #23 | 17 | F |
| #24 | 38 | F |
| #25 | 52 | M |
| #26 | 50 | F |
| #27 | 73 | F |
| #28 | 38 | F |
| #29 | 54 | F |
| #30 | 34 | F |
| #31 | 62 | M |
| #32 | 55 | M |
| #33 | 60 | F |
| #34 | 39 | F |
| #35 | 30 | F |
| #36 (D1) * | 45 | F |
| #37 (D2) * | 20 | F |
| #38 (D3) * | 33 | F |
| Average ±SD | 50.2 ± 16.1 | M:F=14:24 |

Sex: M, male; F, female* Type I diabetics patients

Table S2. Expression of surface antigens and albumin on liver cells analyzed by flow cytometry

| Passage | Early | Mid | Late |
| --- | --- | --- | --- |
| Isotype control | 2.5±2.2 | 1.6±0.5 | 1.2±1.5 |
| CD29 | 99.1±1.7 | 98.5±2.6 | 98.9±0.9 |
| CD31 | 1.4±1.3 | 0.9±0.6 | 0.1±0.2 |
| CD45 | 1.8±1.9 | 1.0±0.4 | 0.1±0.1 |
| CD73 | 92.8±2.9 | 95.0±4.1 | 99.1±1.1 |
| CD90 | 99.6±0.4 | 99.3±1.1 | 99.1±1.1 |
| CD105 | 99.4±0.8 | 99.9±0.1 | 99.5±0.1 |
| Albumin | 19.8±10.5 | 5.2±1.3 | 0.5±0.2 |

Liver cells at early (1-2), mid (6-7), and late (12-14) passages were used. (n=4)

Figure S1. Immunofluorescence for checking the expression of PDX1, NEUROD1, and MAFA in human liver cells. Human liver cells (passage 6) were treated with Ad-CMV-PDX1, Ad-CMV-NEUROD1, and Ad-CMV MAFA with various multiplicities of infection (MOIs) for 2 days.Scale bars denote 200 µm.


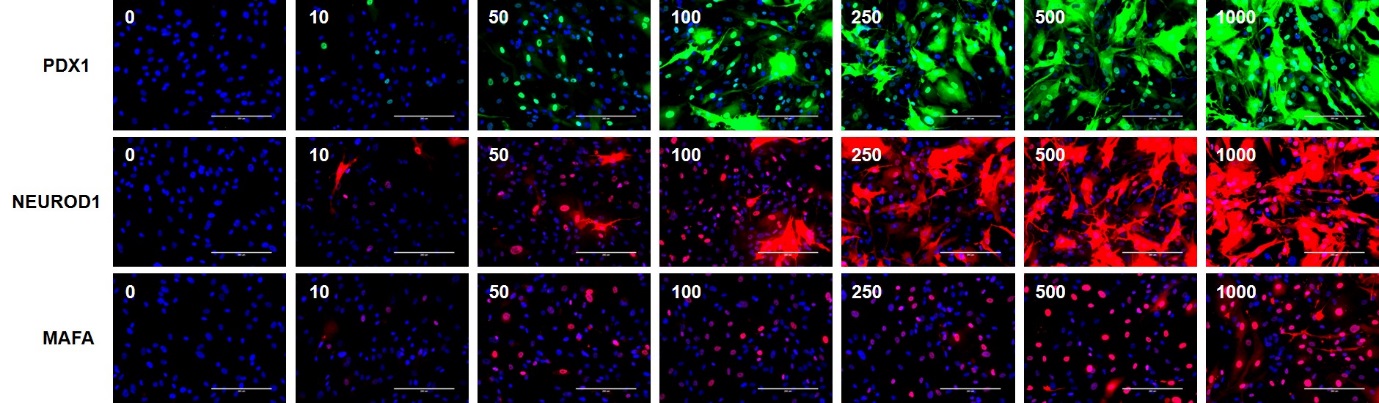


Figure S2. BrdU ELISA cell proliferation assay of liver cells and IPCs on day 2 and day 5 after initial virus transduction. Cells (5×10^3) were plated and allowed to attach for 12 h. BrdU incorporation in cells measured through ELISA (Roche Diagnostics) following 24 h of treatment according to the manufacturer’s directions. There was greater incorporation of BrdU in liver cells and IPCs on day 2 after transduction of initial transcription factor than that in IPCs on day 5. Proliferation was occurring not only in liver cells but also in IPCs in the early stages (day 2) of differentiation, but it was delayed in IPCs on day 5 of insulin production and maturation process.


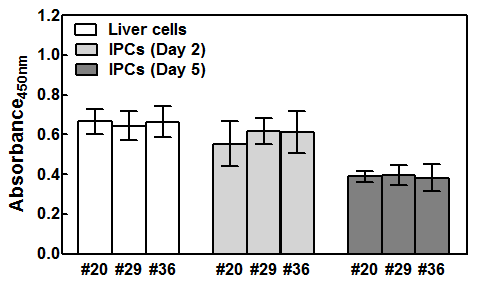

Supplement: Supplementary file 1 — Additional file 1. [file 13287_2020_2080_MOESM1_ESM.docx]
